# Complete mitogenome and phylogenetic analysis of *Oryzias celebensis* (Teleostei: Beloniformes)

**DOI:** 10.1080/23802359.2019.1695550

**Published:** 2019-12-09

**Authors:** Jiangru Ma, Shuisheng Long, Zhongduo Wang

**Affiliations:** Key Laboratory of Aquaculture in South China Sea for Aquatic Economic Animal of Guangdong Higher Education Institutes, Fisheries College, Guangdong Ocean University, Zhanjiang, China

**Keywords:** *Oryzias celebensis*, mitogenome, phylogenetic analysis

## Abstract

The complete *Oryzias celebensis* mitogenome was determined using next-generation sequencing (NGS) method and the resulting data analyzed in this article. The mitochondrial genome was 16,493 base pair (bp) length, and its content and structure were highly homologous to that of other teleostean fishes, including 13 protein-coding genes (PCGs), 2 rRNAs, 22 tRNA, and 1 control region. Among the PCGs, ATG was used as the initiation codon, except for GTG in the COI gene. There were 6 overlapping genes with overlap lengths ranging from 1 to 10 nucleotides (nt), while 10 intergenic regions with a total of 59 nt and a maximum interval of 38 nt between tRNA-Asn and tRNA-Cys were detected during the annotation of this complete mitochondrial DNA.

Celebes medaka, *Oryzias celebensis*, (Teleostei: Beloniformes) is a small fish that can grow up to 4.5 centimeters in length, mainly distributed in rivers and lakes in the Indonesian island of Sulawesi (formerly known as Celebes) and East Timor (Weber 1894). It is a potential fish akin to the freshwater Japanese medaka (*Oryzias latipes*). However, the complete mitogenome genome of *O. celebensis* was still not determined until now.

The mature Celebes medaka were collected from the river in near the national university of Singapore(N1°17′46.81″ E103°45′33.75″). The typical specimen were deposited in the Guangdong Ocean University aquatic museum (O.cele-001). We extracted total RNA using Celebes medaka F1 larvae for sequencing (Wang et al. 2009). According to the sequencing result combined with the information on Genebank database, the complete mitochondrial sequence of the Celebes medaka was obtained, submitted to GenBank and was assigned the accession MN064715. The complete *Oryzias celebensis* mitogenome is 16,493 bp, and its content and structure are highly homologous to those of other teleostean fishes, including 13 protein-coding genes (PCGs), 2 rRNAs, 22 tRNA and 1 control region (Kim and Lee 2004; Liu and Cui 2009). Among the PCGs, except for GTG in the COI gene all genes were initiated with the ATG start codon. There were 6 overlapping genes with overlap lengths ranging from 1 to 10 nucleotides (nt), while ten intergenic regions with a total of 59 nt and a maximum interval of 38 nt between tRNA-Asn and tRNA-Cys.

We constructed the Maximum-Likelihood (ML) consensus tree based on the complete mtDNA gene sequences of Oryzias species (Figure 1). Phylogenetic analysis showed that *O. celebensis* clustered together with *O. sarasinorum* and *O. marmoratus* which is consistent with the previous reports using the nuclear tyrosinase and mitochondrial 12S and 16S rRNA genes (Naruse 1996; Takehana et al. 2005). This indicated the phylogenesis classification of *O. celebensis* is true. The determination of the complete mitogenome sequences provided new molecular data to illuminate the Oryzias evolution.

**Figure 1.**
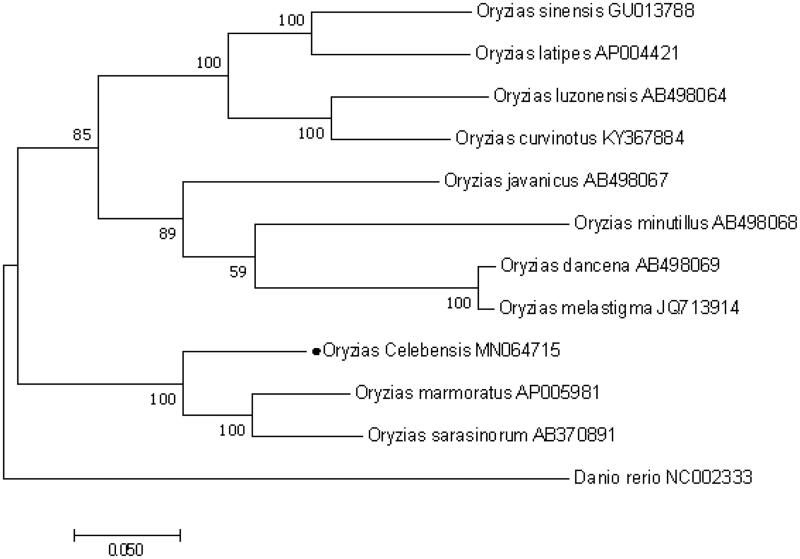
Maximum-Likelihood (ML) consensus tree based on complete mitochondrial DNA (mtDNA) gene sequence of Oryzias species with Danio rerio as an outgroup.

## References

[CIT0001] Kim IC, Lee JS. 2004. The complete mitochondrial genome of the rockfish *Sebastes schlegeli* (Scorpaeniformes, Scorpaenidae). Mol Cells. 17(2):322–328. 15179049

[CIT0002] Liu Y, Cui Z. 2009. The complete mitochondrial genome sequence of the cutlassfish *Trichiurus japonicas* (Perciformes: Trichiuridae): Genome characterization and phylogenetic considerations. Mar Genom. 2(2):133–142. 10.1016/j.margen.2009.07.003 21798182

[CIT0003] Naruse K. 1996. Classification and phylogeny of fishes of the genus Oryzias and its relatives. Fish Biol J Medaka. 8:1–9.

[CIT0004] Takehana Y, Naruse K, Sakaizumi M. 2005. Molecular phylogeny of the medaka fishes genus Oryzias (Beloniformes: Adrianichthyidae) based on nuclear and mitochondrial DNA sequences. Mol Phylogenet Evol. 36(2):417–428. 15955519 10.1016/j.ympev.2005.01.016

[CIT0005] Wang Z, Gerstein M, Snyder M. 2009. RNA-Seq: a revolutionary tool for transcriptomics. Nat Rev Genet. 10(1):57–63. 19015660 10.1038/nrg2484 PMC2949280

[CIT0006] Weber M. 1894. Die Süsswasser-Fische des Indischen Archipels, nebst Bemerkungen über den Ursprung der Fauna von Celebes. Zoologische Ergebnisse einer reise in Niederländisch Ost-Indien. 3:405–476.

